# Supplementary material for: Role of community pharmacists in the safe and effective use of complementary and alternative medicine in the Middle East: A scoping review
Source: PLoS One. 2025 Sep 26;20(9):e0332932. doi: 10.1371/journal.pone.0332932 (PMC12469160; doi:10.1371/journal.pone.0332932)
Supplement: S1 Appendix — (DOCX) [file pone.0332932.s001.docx]

S1 Appendix: Search Strategy

| Search | Query |
| --- | --- |
| 1 | title:(pharmacist*) OR ab:(pharmacist*) OR subject:(pharmacist*) |
| 2 | (title:("chinese medicine*" OR "chinese medication*" OR "chinese remed*" OR “horticultural therap*” OR "pharmaceutical plant*" OR "healing plant*" OR micronutrient* OR multivitamin*) OR ab:("chinese medicine*" OR "chinese medication*" OR "chinese remed*" OR “horticultural therap*” OR "pharmaceutical plant*" OR "healing plant*" OR micronutrient* OR multivitamin*) OR subject:("chinese medicine*" OR "chinese medication*" OR "chinese remed*" OR “horticultural therap*” OR "pharmaceutical plant*" OR "healing plant*" OR micronutrient* OR multivitamin*)) OR (title:(“Arabic remed*” OR "Islamic medicine*" OR "Islamic medication*" OR "Islamic remed*" OR phytotherap* OR “medicinal plant*” OR “plant extract*” ) OR ab:(“Arabic remed*” OR "Islamic medicine*" OR "Islamic medication*" OR "Islamic remed*" OR phytotherap* OR “medicinal plant*” OR “plant extract*” ) OR subject:(“Arabic remed*” OR "Islamic medicine*" OR "Islamic medication*" OR "Islamic remed*" OR phytotherap* OR “medicinal plant*” OR “plant extract*” )) OR (title:(“naturopathic medication*” OR naturopathy OR “health* food*” OR “natural product*” OR botanical* OR probiotic* OR “Arabic medicine*” OR “Arabic medication*” ) OR ab:(“naturopathic medication*” OR naturopathy OR “health* food*” OR “natural product*” OR botanical* OR probiotic* OR “Arabic medicine*” OR “Arabic medication*” ) OR subject:(“naturopathic medication*” OR naturopathy OR “health* food*” OR “natural product*” OR botanical* OR probiotic* OR “Arabic medicine*” OR “Arabic medication*” )) OR (title:(“traditional health” OR "traditional treatment*" OR vitamin* OR mineral* OR herb OR herbs OR herbal OR supplement* OR “naturopathic medicine*”) OR ab:(“traditional health” OR "traditional treatment*" OR vitamin* OR mineral* OR herb OR herbs OR herbal OR supplement* OR “naturopathic medicine*”) OR subject:(“traditional health” OR "traditional treatment*" OR vitamin* OR mineral* OR herb OR herbs OR herbal OR supplement* OR “naturopathic medicine*”)) OR (title:("integrative health" OR "integrative treatment*" OR "traditional therap*" OR "traditional medicine*" OR "traditional medication*" OR "traditional remed*") OR ab:("integrative health" OR "integrative treatment*" OR "traditional therap*" OR "traditional medicine*" OR "traditional medication*" OR "traditional remed*") OR subject:("integrative health" OR "integrative treatment*" OR "traditional therap*" OR "traditional medicine*" OR "traditional medication*" OR "traditional remed*")) OR (title:(“alternative health” OR “alternative treatment*" OR “integrative therap*” OR “integrative medicine*” OR “integrative medication*” OR “integrative remed*” ) OR ab:(“alternative health” OR “alternative treatment*" OR “integrative therap*” OR “integrative medicine*” OR “integrative medication*” OR “integrative remed*” ) OR subject:(“alternative health” OR “alternative treatment*" OR “integrative therap*” OR “integrative medicine*” OR “integrative medication*” OR “integrative remed*” )) OR (title:("complementary treatment*" OR “alternative therap*” OR “alternative medicine*” OR “alternative medicat*” OR “alternative remed*” ) OR ab:("complementary treatment*" OR “alternative therap*” OR “alternative medicine*” OR “alternative medicat*” OR “alternative remed*” ) OR subject:("complementary treatment*" OR “alternative therap*” OR “alternative medicine*” OR “alternative medicat*” OR “alternative remed*” )) OR (title:(“complementary therap*” OR “complementary medicine*” OR “complementary medication*” OR “complementary remed*” OR “complementary health” ) OR ab:(“complementary therap*” OR “complementary medicine*” OR “complementary medication*” OR “complementary remed*” OR “complementary health” ) OR subject:(“complementary therap*” OR “complementary medicine*” OR “complementary medication*” OR “complementary remed*” OR “complementary health” )) OR (title:(“complementary and alternative remed*” OR “complementary and alternative health” OR "complementary and alternative treatment*") OR ab:(“complementary and alternative remed*” OR “complementary and alternative health” OR "complementary and alternative treatment*") OR subject:(“complementary and alternative remed*” OR “complementary and alternative health” OR "complementary and alternative treatment*")) OR (title:(CAM OR “complementary and alternative therap*” OR “complementary and alternative medicine*” OR “complementary and alternative medication*”) OR ab:(CAM OR “complementary and alternative therap*” OR “complementary and alternative medicine*” OR “complementary and alternative medication*”) OR subject:(CAM OR “complementary and alternative therap*” OR “complementary and alternative medicine*” OR “complementary and alternative medication*”)) |
| 3 | title:(bahrain* OR bahrein* OR cyprus OR cypriot* OR egypt* OR iran* OR persia* OR iraq* OR Irak* OR Israel* OR jordan* OR kuwait* OR koweit* OR kuweit* OR lebanon OR lebanese OR liban OR libanaise* OR oman* OR palestin* OR gaza* OR "west bank" OR qatar* OR saudi* OR saoudi* OR KSA OR syria* OR syrie* OR turkey OR turkish OR turks OR turk OR "united arab emirates" OR UAE OR "abu dhabi" OR "abu dabi" OR dubai OR ajman OR fujaira* OR sharja* OR khaima* OR qaiwain OR quwain OR yemen* OR "middle east*" OR arab OR arabic OR arabia OR arabs OR "near east*" OR levant* OR MENA OR EMRO OR gulf* OR "east* mediterranean") OR ab:(bahrain* OR bahrein* OR cyprus OR cypriot* OR egypt* OR iran* OR persia* OR iraq* OR Irak* OR Israel* OR jordan* OR kuwait* OR koweit* OR kuweit* OR lebanon OR lebanese OR liban OR libanaise* OR oman* OR palestin* OR gaza* OR "west bank" OR qatar* OR saudi* OR saoudi* OR KSA OR syria* OR syrie* OR turkey OR turkish OR turks OR turk OR "united arab emirates" OR UAE OR "abu dhabi" OR "abu dabi" OR dubai OR ajman OR fujaira* OR sharja* OR khaima* OR qaiwain OR quwain OR yemen* OR "middle east*" OR arab OR arabic OR arabia OR arabs OR "near east*" OR levant* OR MENA OR EMRO OR gulf* OR "east* mediterranean") OR subject:(bahrain* OR bahrein* OR cyprus OR cypriot* OR egypt* OR iran* OR persia* OR iraq* OR Irak* OR Israel* OR jordan* OR kuwait* OR koweit* OR kuweit* OR lebanon OR lebanese OR liban OR libanaise* OR oman* OR palestin* OR gaza* OR "west bank" OR qatar* OR saudi* OR saoudi* OR KSA OR syria* OR syrie* OR turkey OR turkish OR turks OR turk OR "united arab emirates" OR UAE OR "abu dhabi" OR "abu dabi" OR dubai OR ajman OR fujaira* OR sharja* OR khaima* OR qaiwain OR quwain OR yemen* OR "middle east*" OR arab OR arabic OR arabia OR arabs OR "near east*" OR levant* OR MENA OR EMRO OR gulf* OR "east* mediterranean") OR gl:(bahrain* OR bahrein* OR cyprus OR cypriot* OR egypt* OR iran* OR persia* OR iraq* OR Irak* OR Israel* OR jordan* OR kuwait* OR koweit* OR kuweit* OR lebanon OR lebanese OR liban OR libanaise* OR oman* OR palestin* OR gaza* OR "west bank" OR qatar* OR saudi* OR saoudi* OR KSA OR syria* OR syrie* OR turkey OR turkish OR turks OR turk OR "united arab emirates" OR UAE OR "abu dhabi" OR "abu dabi" OR dubai OR ajman OR fujaira* OR sharja* OR khaima* OR qaiwain OR quwain OR yemen* OR "middle east*" OR arab OR arabic OR arabia OR arabs OR "near east*" OR levant* OR MENA OR EMRO OR gulf* OR "east* mediterranean") |
