# Supplementary material for: Role of community pharmacists in the safe and effective use of complementary and alternative medicine in the Middle East: A scoping review
Source: PLoS One. 2025 Sep 26;20(9):e0332932. doi: 10.1371/journal.pone.0332932 (PMC12469160; doi:10.1371/journal.pone.0332932)
Supplement: S2 Appendix — (DOCX) [file pone.0332932.s002.docx]

S2 Appendix. Data Charting form

**Role/responsibilities of community pharmacists in CAM**

1. **General information about the article**
   1. Article ID number
   2. Code Name (person who enters the data)
   3. Title of the study
   4. Language of publication
      - 1=English
      - 2=Arabic
      - 3=French
      - 4=Turkish
      - 5=Persian
      - 6=Hebrew
   5. Journal Name (full)
   6. Year of publication of the document type (date of print)
   7. Journal IF current
   8. Citation number
   9. Affiliation of all the authors (select all that apply 1=Yes and 0=No)
      1. Academic institutions
      2. NGO
      3. Governmental agencies
      4. Hospital
      5. Other-Specify
         1. Specify (string)
      6. Unknown
   10. 1^st^ Affiliation of the **corresponding** author (only the name of the institution the author works in) (string)
       1. 2^nd^ Affiliation of the **corresponding** author (only the name of the institution the author works in) (string)
   11. Name of the **corresponding author** (written as family name then the first letter of the first name and middle name only if provided in the paper e.g.: Sibai AM, Haida M, Fouad MF
   12. Country of the 1^st^ affiliating institution of the **corresponding author**

1=Bahrain; 2=Egypt; 3=Iran (OR Persia); 4=Iraq; 5=Israel; 6=Jordan; 7=Kuwait; 8=Lebanon; 9=Oman; 10=Palestine (OR Gaza OR West Bank); 11=Qatar; 12=Saudi Arabia (OR KSA); 13=Syria; 14=Turkey; 15=United Arab Emirates (OR UAE or the individual states); 16=Yemen; Cyprus=17

- - 1. Country of the 2^nd^ affiliating institution of the **corresponding author**

1=Bahrain; 2=Egypt; 3=Iran (OR Persia); 4=Iraq; 5=Israel; 6=Jordan; 7=Kuwait; 8=Lebanon; 9=Oman; 10=Palestine (OR Gaza OR West Bank); 11=Qatar; 12=Saudi Arabia (OR KSA); 13=Syria; 14=Turkey; 15=United Arab Emirates (OR UAE or the individual states); 16=Yemen’; Cyprus=17

- 1. 1^st^ Affiliation of the **Last author (if different from corresponding)** author (only the name of the institution the author works in) (string)
     1. 2^nd^ Affiliation of the **Last author (if different from corresponding)** author (only the name of the institution the author works in) (string)
  2. Name of the **Last author (if different from corresponding)** (written as family name then the first letter of the first name and middle name only if provided in the paper e.g.: Sibai AM, Haida M, Fouad MF
  3. Country of the 1^st^ affiliating institution of the **Last author (if different from corresponding)**

1=Bahrain; 2=Egypt; 3=Iran (OR Persia); 4=Iraq; 5=Israel; 6=Jordan; 7=Kuwait; 8=Lebanon; 9=Oman; 10=Palestine (OR Gaza OR West Bank); 11=Qatar; 12=Saudi Arabia (OR KSA); 13=Syria; 14=Turkey; 15=United Arab Emirates (OR UAE or the individual states); 16=Yemen; Cyprus=17.

- - 1. Country of the 2^nd^ affiliating institution of the **Last author (if different from corresponding)**

1=Bahrain; 2=Egypt; 3=Iran (OR Persia); 4=Iraq; 5=Israel; 6=Jordan; 7=Kuwait; 8=Lebanon; 9=Oman; 10=Palestine (OR Gaza OR West Bank); 11=Qatar; 12=Saudi Arabia (OR KSA); 13=Syria; 14=Turkey; 15=United Arab Emirates (OR UAE or the individual states); 16=Yemen; Cyprus=17.

- 1. 1^st^ Affiliation of the **first author** (only the name of the institution the author works in) (string)
     1. 2^nd^ Affiliation of the **first author** (only the name of the institution the author works in) (string)
  2. Name of the **first author** (written as family name then the first letter of the first name and middle name only if provided in the paper e.g.: Sibai AM, Haida M, Fouad MF
  3. Country of the 1^st^ affiliating institution of the **first author**

1=Bahrain; 2=Egypt; 3=Iran (OR Persia); 4=Iraq; 5=Israel; 6=Jordan; 7=Kuwait; 8=Lebanon; 9=Oman; 10=Palestine (OR Gaza OR West Bank); 11=Qatar; 12=Saudi Arabia (OR KSA); 13=Syria; 14=Turkey; 15=United Arab Emirates (OR UAE or the individual states); 16=Yemen; Cyprus=17

- - 1. Country of the 2^nd^ affiliating institution of the **first author**

1=Bahrain; 2=Egypt; 3=Iran (OR Persia); 4=Iraq; 5=Israel; 6=Jordan; 7=Kuwait; 8=Lebanon; 9=Oman; 10=Palestine (OR Gaza OR West Bank); 11=Qatar; 12=Saudi Arabia (OR KSA); 13=Syria; 14=Turkey; 15=United Arab Emirates (OR UAE or the individual states); 16=Yemen; Cyprus=17

- 1. Collaboration of authors between countries

1=No collaboration, all authors same country

2=Collaboration between Middle East countries (specify)

3=Collaboration between ME and non-ME-countries (specify)

4=Others (specify)

- - 1. Specify collaboration between ME countries (string)
    2. Specify collaborations between ME and None-ME countries (string)
    3. Specify Other collaborations (sting)
    4. If all authors same country are they collaborating with different institution? (Yes=1, No=0)
  1. Study Design
     - 1=Case report
     - 2=Case series
     - **3=Cross-sectional study**
     - 4=Case-control study
     - 5=Cohort study
     - 6=RCT
     - **7=Systematic Review/Meta-analysis**
     - **8=Review (Literature review)**
     - **9=Focus Group Discussions/KEY Informant Interviews**
     - 10=Laboratory/Basic Science studies/Basic Science (animal-based/pathological/in-vivo in-vitro studies) [RCT phase 0]
     - 11=N/A

1. **Study population (Who)**
   1. Sample size (numeric) [include the total sample]
2. **Study characteristics**
   1. Type of data collected (Select all that apply) (1=Yes and 0=No)
      1. Qualitative (go to D)
      2. Quantitative
      3. Mixed methods
   2. Specify the name(s) of the Funding organization (string)
      1. Funding grouped

1=Government

2=Private/industry/academic

3=NGO

4=Other

5=unknown

6=more than one

7=not available

8=No Funding

- 1. Was the study IRB approved? (1=Yes 0=No (IRB was not sought) 2= Not mentioned, 3=N/A)

1. **In case the data collected was qualitative**
   1. Type of qualitative study (select all that apply) (1=Yes and 2=No)
      1. Focus group discussions
      2. Key informant interviews
      3. Focus group discussions and key informant interviews
      4. Other, specify (string)
2. **Pharmacy section**
   1. Type of study participants (select all that applies Yes=1, No=0)
      1. Pharmacy students
      2. Community pharmacy
      3. Consumers
      4. Medical/hospital based pharmacy
      5. N/A
      6. others (specify)
         1. Other Specify (string)
   2. Aim (pull out from abstract)
   3. Inclusion criteria
   4. Results (enter as string)-pull out from abstract (result and conclusion)
   5. Reported findings related to role of Pharmacy? (ex: this study reported inadequate knowledge about safe use of CAM)
   6. Type of CAM in the study (string)
   7. How was CAM defined (definition based on what criteria ex ICCM….)
   8. Was the questionnaire validated (Yes=1, No=0, 2=N/A)
   9. Was the questionnaire pilot tested (Yes=1, No=0, 2=N/A)
   10. Recommendations provided on the role of CP on CAM_________________
   11. Recommendations given for the pharmacy curriculum or continue education program________
   12. Other healthcare providers in the study? (select all that applies Yes=1, No=0)
       1. Physicians
       2. Herbalists
       3. Others: Specify
   13. Responsibilities of the pharmacists regarding CAM.
       1. Acknowledge the use
       2. To be knowledgeable about CAM products
       3. Ensure safe use of CAM
       4. Document the use of CAM
       5. Report adverse reactions related to CAM
       6. To educate about CAM
       7. To collaborate with other healthcare professionals
       8. Others: Specify
   14. Is the main Aim of the article about CAM and Pharmacy Role

1=Yes

0=No, just a minor part

- 1. Notes to the reviewer (add notes to the second reviewed ex: double check A.20, I was not sure about question A.19. etc.)
  2. Additional notes related to the article
