# Supplementary material for: Role of community pharmacists in the safe and effective use of complementary and alternative medicine in the Middle East: A scoping review
Source: PLoS One. 2025 Sep 26;20(9):e0332932. doi: 10.1371/journal.pone.0332932 (PMC12469160; doi:10.1371/journal.pone.0332932)
Supplement: S3 Appendix — (DOCX) [file pone.0332932.s003.docx]

**S3 Appendix.** Included studies and their countries.

| **Reference** | **Country** |
| --- | --- |
| L. Alnaim et al., “Pharmacist’s Knowledge and Attitudes towards Complementary and Alternative Medicine in Saudi Arabia: A Cross-Sectional Study,” *Advances in Integrative Medicine* 5, no. 3 (2018): 96–102, Scopus, https://doi.org/10.1016/j.aimed.2018.01.003. | Saudi Arabia |
| Ali Alshahrani, “Knowledge, Attitudes, and Practice of Community Pharmacists towards Providing Counseling on Vitamins, and Nutritional Supplements in Saudi Arabia,” *AIMS Public Health* 7, no. 3 (2020): 697. | Saudi Arabia |
| A. S. Alzahrani et al., “Views of Healthcare Professionals on Complementary and Alternative Medicine Use by Patients with Diabetes: A Qualitative Study,” *BMC COMPLEMENTARY MEDICINE AND THERAPIES* 24, no. 1 (2024), https://doi.org/10.1186/s12906-024-04385-6. | Saudi Arabia |
| H. M. Al-Yousef et al., “Knowledge, Attitude and Dispensing Practice of the Pharmacists Related to Complementary Alternative Medicines in the Riyadh Region of Saudi Arabia: A Cross-Sectional Descriptive Study,” *BMC HEALTH SERVICES RESEARCH* 22, no. 1 (2022), https://doi.org/10.1186/s12913-022-08932-0. | Saudi Arabia |
| K.F. Alshammari et al., “Assessment of Knowledge about Traditional Medicine Reveals Overuse as a Potential Risk for Aggravating COVID-19 and Underlying Diseases in Geriatrics and Women’s Health in the Saudi Population,” *Clinics and Practice* 12, no. 3 (2022): 363–73, Scopus, https://doi.org/10.3390/clinpract12030041. | Saudi Arabia |
| Muazzam M. Sheriff et al., “A Cross-Sectional Study on the Awareness and Practice of the Use of Supplemental Vitamin C, Arginine, and Zinc in Managing Wounds Among Healthcare Workers in Saudi Arabia,” *Cureus* 15, no. 12 (2023): e51235, https://dx.doi.org/10.7759/cureus.51235. | Saudi Arabia |
| A. A. Alrasheedy, “Pharmaceutical Activities, Services, Medicines, and Products at Community Pharmacies in the Qassim Region of Saudi Arabia: Findings and Implications for the Future,” *CUREUS JOURNAL OF MEDICAL SCIENCE* 16, no. 8 (2024), https://doi.org/10.7759/cureus.67957. | Saudi Arabia |
| Saad S. Alqahtani et al., “Knowledge, Attitude, and Practice Regarding Vitamin D and Dietary Supplements Use: A Community Pharmacy Based Cross-Sectional Study in Jazan, Saudi Arabia,” *Current Topics in Nutraceutical Research* 20.2 (2022). | Saudi Arabia |
| M.H. Mahnashi, “Knowledge, Attitude, Practice, and the Perceived Barriers with Respect to the Use of Herbal Medicines,” *Current Topics in Nutraceutical Research* 19, no. 1 (2021): 29–35, Scopus, https://doi.org/10.37290/ctnr2641-452x.19:29-35. | Saudi Arabia |
| S. M. Alshahrani, “Community Pharmacists’ Knowledge, Attitudes, and Barriers Toward Prescribing Probiotics in Aseer Region, Saudi Arabia,” *CURRENT TOPICS IN NUTRACEUTICAL RESEARCH* 22, no. 2 (2024): 612–16, https://doi.org/10.37290/ctnr2641-452X.22:612-616. | Saudi Arabia |
| Alqahtani et al., “Knowledge, Attitude, and Practice Regarding Vitamin D and Dietary Supplements Use: A Community Pharmacy Based Cross-Sectional Study in Jazan, Saudi Arabia.” | Saudi Arabia |
| K. M. Alkharfy, “Community Pharmacists’ Knowledge, Attitudes and Practices towards Herbal Remedies in Riyadh, Saudi Arabia.,” *Eastern Mediterranean Health Journal* 16, no. 9 (2010): 988–93. | Saudi Arabia |
| Abdulrhman Alsayari et al., “Community Pharmacists’ Knowledge, Attitudes, and Practice of Herbal Medicines in Asir Region, Kingdom of Saudi Arabia,” *Evidence‐Based Complementary and Alternative Medicine* 2018, no. 1 (2018): 1568139. | Saudi Arabia |
| Ahmed Y Aldryhim et al., “Factors That Facilitate Reporting of Adverse Drug Reactions by Pharmacists in Saudi Arabia.,” *Expert Opinion on Drug Safety* (England) 18, no. 8 (2019): 745–52, Ovid MEDLINE(R) <2019>, https://doi.org/10.1080/14740338.2019.1632287. | Saudi Arabia |
| S. B. Loni et al., “Prevalence of Self-Medication and Associated Factors among Female Students of Health Science Colleges at Majmaah University: A Cross-Sectional Study,” *FRONTIERS IN PUBLIC HEALTH* 11 (February 2023), https://doi.org/10.3389/fpubh.2023.1090021. | Saudi Arabia |
| A. H. Alghadir et al., “Attitude, Beliefs, and Use of Herbal Remedies by Patients in the Riyadh Region of Saudi Arabia,” *HEALTHCARE* 10, no. 5 (2022), https://doi.org/10.3390/healthcare10050907. | Saudi Arabia |
| M.E. Elrggal et al., “Dispensing Practices for Weight Management Products in Eastern Saudi Arabia: A Survey of Community Pharmacists,” *International Journal of Environmental Research and Public Health* 18, no. 24 (2021), Scopus, https://doi.org/10.3390/ijerph182413146. | Saudi Arabia |
| E. Alshammari, “Navigating Natural Products Resources: Simulated Session for Pharmacy Students,” *International Journal of Pharmaceutical Research* 12 (2020): 3559–64, Scopus, https://doi.org/10.31838/ijpr/2020.SP2.422. | Saudi Arabia |
| Amal K. Suleiman, “Attitudes and Beliefs of Consumers of Herbal Medicines in Riyadh, Saudi Arabia,” *Journal of Community Medicine & Health Education* 4, no. 2 (2014): 2, https://doi.org/10.4172/2161-0711.1000269. | Saudi Arabia |
| Abdullah M. AlBedah et al., “Knowledge and Attitude of Health Professionals in the Riyadh Region, Saudi Arabia, toward Complementary and Alternative Medicine,” *Journal of Family & Community Medicine* 19, no. 2 (2012): 93–99, https://doi.org/10.4103/2230-8229.98290. | Saudi Arabia |
| S. A. Alhubail et al., “Healthcare Professionals and Undergraduate Students’ Knowledge Toward Drug-Food Interactions in the Eastern Region of Saudi Arabia,” *JOURNAL OF MULTIDISCIPLINARY HEALTHCARE* 16 (2023): 2883–92, https://doi.org/10.2147/JMDH.S419544. | Saudi Arabia |
| NJ Ahmed and MF Khan, “Community Pharmacists’ Knowledge, Attitudes and Practices towards the Use of Herbal Remedies,” *JOURNAL OF PHARMACEUTICAL RESEARCH INTERNATIONAL* 31, no. 6 (2019), WOS:000497734600001, https://doi.org/10.9734/JPRI/2019/v31i630316. | Saudi Arabia |
| Sherihan A Ghosn et al., “Community Pharmacist’s Knowledge, Attitude, and Practices towards Vitamin Supplements in Al-Khobar Region, Saudi Arabia: A Descriptive Cross-Sectional Study.,” *Journal of Pharmacy & Bioallied Sciences* (India), Erratum in: J Pharm Bioallied Sci. 2020 Jan-Mar;12(1):82 PMID: 32801605 [https://www.ncbi.nlm.nih.gov/pubmed/32801605], vol. 11, no. 4 (2019): 333–40, Ovid MEDLINE(R) PubMed-not-MEDLINE <2017 to 2019>, https://doi.org/10.4103/jpbs.JPBS_99_19. | Saudi Arabia |
| A. Bajuayfir et al., “Evaluation of Current Community Pharmacist Practice in Saudi Arabia: A Cross-Sectional Study from Patients’ Perspective (PART I),” *JOURNAL OF RESEARCH IN PHARMACY PRACTICE* 12, no. 1 (2023): 15–20, https://doi.org/10.4103/jrpp.jrpp_12_23. | Saudi Arabia |
| MN Al-Arifi et al., “Evaluation of Knowledge of Health Care Professionals on Warfarin Interactions with Drug and Herbal Medicines,” *PAKISTAN JOURNAL OF MEDICAL SCIENCES* 32, no. 1 (2016): 229–33, WOS:000369318000049. | Saudi Arabia |
| A. Bahha et al., “Awareness of Natural Herbs’ Effect on Blood Pressure among the Western Region of Saudi Arabia Population: A Cross-Sectional Study,” *PHARMACOGNOSY RESEARCH* 14, no. 4 (2022): 461–67, https://doi.org/10.5530/pres.14.4.67. | Saudi Arabia |
| M. Tobaiqy et al., “Community Pharmacists’ Knowledge, Attitudes and the Perceived Safety and Effectiveness of Melatonin Supplements: A Cross-Sectional Survey,” *PHARMACY* 11, no. 5 (2023), https://doi.org/10.3390/pharmacy11050147. | Saudi Arabia |
| Sultan M Alshahrani, “Assessment of Knowledge, Attitudes, and Practice of Community Pharmacists Regarding Weight Reduction Agents and Supplements in Aseer Region, Saudi Arabia.,” *Risk Management and Healthcare Policy* (England) 13, no. 101566264 (2020): 347–53, Ovid MEDLINE(R) PubMed-not-MEDLINE <2020>, https://doi.org/10.2147/RMHP.S234818. | Saudi Arabia |
| Noha M Zaki and Ahmed A Albarraq, “Use, Attitudes and Knowledge of Medications among Pregnant Women: A Saudi Study.,” *Saudi Pharmaceutical Journal : SPJ : The Official Publication of the Saudi Pharmaceutical Society* (Saudi Arabia) 22, no. 5 (2014): 419–28, Ovid MEDLINE(R) PubMed-not-MEDLINE <2013 to 2016>, https://doi.org/10.1016/j.jsps.2013.09.001. | Saudi Arabia |
| Ziyad Alrabiah et al., “Knowledge of Community Pharmacists about the Risks of Medication Use during Pregnancy in Central Region of Saudi Arabia,” *Saudi Pharmaceutical Journal* 25, no. 7 (2017): 1093–96. | Saudi Arabia |
| Mohamed N Al-Arifi, “Availability and Needs of Herbal Medicinal Information Resources at Community Pharmacy, Riyadh Region, Saudi Arabia.,” *Saudi Pharmaceutical Journal : SPJ : The Official Publication of the Saudi Pharmaceutical Society* (Saudi Arabia) 21, no. 4 (2013): 351–60, Ovid MEDLINE(R) PubMed-not-MEDLINE, https://doi.org/10.1016/j.jsps.2012.11.004. | Saudi Arabia |
| S. D. Babelghaith et al., “The Use of Complementary and Alternative Medicine for Functional Gastrointestinal Disorders among the Saudi Population,” *SAUDI PHARMACEUTICAL JOURNAL* 32, no. 6 (2024), https://doi.org/10.1016/j.jsps.2024.102084. | Saudi Arabia |
| F. El-Gamal et al., “Knowledge, Attitude, and Practice towards Over-the-Counter Drugs (OTC) Use among Adult Population in Jeddah, Saudi Arabia,” *WORLD FAMILY MEDICINE* 20, no. 6 (2022): 84–90, https://doi.org/10.5742/MEWFM.2022.9525056. | Saudi Arabia |
| B. Jalil et al., “Herbal Supplements in Jordan: A Cross-Sectional Survey of Pharmacists’ Perspectives and Knowledge,” *BMJ OPEN* 12, no. 7 (2022), https://doi.org/10.1136/bmjopen-2021-057405. | Jordan |
| D. Jaber and A. Alhusban, “Long-Term Complications of COVID-19 in Jordan: A Study on Persisting Symptoms and Management,” *CLINICAL EPIDEMIOLOGY AND GLOBAL HEALTH* 28 (August 2024), https://doi.org/10.1016/j.cegh.2024.101627. | Jordan |
| Kanar Sweiss et al., “Jordanian Use of and Beliefs Concerning the Efficacy of Medicinal Plants: A Cross-Sectional Study,” *Cureus* 15, no. 4 (2023): e37494, https://dx.doi.org/10.7759/cureus.37494. | Jordan |
| M. Ababneh et al., “Evaluation of Jordanian Healthcare Providers’ Knowledge, Attitudes, and Practice Patterns towards Probiotics,” *Expert Review of Pharmacoeconomics and Outcomes Research* 20, no. 1 (2020): 93–97, Scopus, https://doi.org/10.1080/14737167.2019.1609354. | Jordan |
| S. F. Al-Shatnawi et al., “Healthcare Providers’ Perspectives Toward the Integration of over the Counter Supplements During COVID-19 Pandemic: A Cross-Sectional Study from Jordan,” *INQUIRY-THE JOURNAL OF HEALTH CARE ORGANIZATION PROVISION AND FINANCING* 59 (April 2022), https://doi.org/10.1177/00469580221095825. | Jordan |
| Alaa Saadeh et al., “Assessment of Jordanian Community Pharmacists’ Confidence to Provide Health Services and Advice on Self-Medication for Women during Pregnancy and Breastfeeding,” *The International Journal of Pharmacy Practice* 31, no. 2 (2023): 183–89, https://dx.doi.org/10.1093/ijpp/riad003. | Jordan |
| R. M. Daghash et al., “Evaluation of Pharmacotherapy Standards During Pregnancy Among Jordanian Pharmacy Colleges Graduates,” *Jordan Journal of Pharmaceutical Sciences* 16, no. 3 (2023): 607–20, https://doi.org/10.35516/jjps.v16i3.972. | Jordan |
| Yousef Khader et al., “Knowledge and Attitudes of Lay Public, Pharmacists, and Physicians toward the Use of Herbal Products in North Jordan.,” *Journal of Alternative and Complementary Medicine (New York, N.Y.)* (United States) 14, no. 10 (2008): 1186–87, Ovid MEDLINE(R) <2008 to 2009>, https://doi.org/10.1089/acm.2008.0282. | Jordan |
| Isra Dmour et al., “Self-Care Interventions among the Jordanians during the COVID-19 Lockdown: A Cross-Sectional Study of Community Pharmacists’ Observations,” *Journal of Applied Pharmaceutical Science* 13, no. 8 (2023): 212–22, https://doi.org/10.7324/japs.2023.143803. | Jordan |
| O. S. Gammoh et al., “Valerian: An Underestimated Anxiolytic in the Community Pharmacy?,” in *Journal of Herbal Medicine*, vol. 6, no. 4, München, Elsevier GmbH, 2016. | Jordan |
| R. M. Darwish et al., “Users’ Knowledge and Self Medications in Relation to Gastric Problems among Adults in a Middle Income Country; Jordan,” *Journal of Substance Use* 27, no. 5 (2022): 470–74, https://doi.org/10.1080/14659891.2021.1961323. | Jordan |
| Abdallah F Omeish et al., “Hospital-Based Study on the Use of Herbal Medicine in Patients with Coronary Artery Disease in Jordan.,” *JPMA. The Journal of the Pakistan Medical Association* (Pakistan) 61, no. 7 (2011): 683–87, Ovid MEDLINE(R) <2010 to 2011>. | Jordan |
| NAKY Younis, “The Prevalence, Attitude and Awareness of Herbal Medicine Products Use among Pharmacy Practitioner in Jordan.,” in *Pharmacognosy Journal*, vol. 11, no. 5, Bangalore, Phcog.Net, 2019. | Jordan |
| Sireen A Shilbayeh, “Exploring Knowledge and Attitudes towards Counselling about Vitamin Supplements in Jordanian Community Pharmacies,” *Pharmacy Practice* 9, no. 4 (2011): 242. | Jordan |
| M. A. Alameri et al., “Awareness, Knowledge, and Behaviour of Jordanian Public Regarding Misuse and Overuse of Proton Pump Inhibitors,” *Pharmacy Practice* 22, no. 2 (2024), https://doi.org/10.18549/PharmPract.2024.2.2870. | Jordan |
| A. S. Jarab et al., “Practice, Competency and Attitude toward Weight Management Service and the Barriers for Its Implementation in the Community Pharmacy Setting,” *Pharmacy Practice* 22, no. 2 (2024), https://doi.org/10.18549/PharmPract.2024.2.2937. | Jordan |
| R. I. Nassar et al., “The Battle against COVID-19 in Jordan: A Cross-Sectional Study Assessing the Experience of Jordanians Who Have Been Infected with COVID-19,” *PHARMACY PRACTICE-GRANADA* 21, no. 1 (2023), https://doi.org/10.18549/PharmPract.2023.1.2791. | Jordan |
| Mayyada Wazaify et al., “Societal Perspectives on the Role of Community Pharmacists and Over-the-Counter Drugs in Jordan,” *Pharmacy World & Science: PWS* 30, no. 6 (2008): 884–91, https://doi.org/10.1007/s11096-008-9244-1. | Jordan |
| Fatima Alnaimat et al., “Rates, Patterns, and Predictors of Complementary Medicine Use among Patients with Musculoskeletal Diseases,” *PloS One* 18, no. 6 (2023): e0287337, https://dx.doi.org/10.1371/journal.pone.0287337. | Jordan |
| N. Bulatova et al., “Use of Traditional and Complementary Medicine for COVID 19 Prophylaxis among Healthcare Professionals and Students in Jordan: A Cross-Sectional Study,” *PLoS ONE* 17, no. 10 October (2022), https://doi.org/10.1371/journal.pone.0276015. | Jordan |
| Ahmed Maslat et al., “DRUG MISUSE AND SELF-MEDICATION AMONG PHARMACY STUDENTS IN JORDAN,” *Polski Merkuriusz Lekarski : Organ Polskiego Towarzystwa Lekarskiego* 51, no. 5 (2023): 464–74, https://dx.doi.org/10.36740/Merkur202305104. | Jordan |
| A. A. Suleiman et al., “Prevalence of Vitamin-Mineral Supplement Use among Jordan University Students.,” *Saudi Medical Journal* 29, no. 9 (2008): 1326–31. | Jordan |
| S. H. Thiab et al., “Medications and Natural Products Used in Jordan for Prevention or Treatment of COVID-19 Infection during the Second Wave of the Pandemic: A Cross-Sectional Online Survey,” *SAUDI PHARMACEUTICAL JOURNAL* 30, no. 6 (2022): 856–62, https://doi.org/10.1016/j.jsps.2022.03.006. | Jordan |
| Reem A. Issa and Iman A. Basheti, *Herbal Products Use Among Chronic Patients and Its Impact on Treatments Safety and Efficacy: A Clinical Survey in the Jordanian Field*, no. 12 (2017): 32–44. | Jordan |
| Eyad Qunaibi et al., *Effect of Divergence in Patients’ Socioeconomic Background on Their Perspective of the Role of the Community Pharmacist in Amman, Jordan*, Pharmacotherapy Group, Faculty of Pharmacy, University of Benin, Benin City, Nigeria, May 13, 2013, http://hdl.handle.net/1807/60939. | Jordan |
| I.A. Basheti et al., “Opinions of Pharmacists and Herbalists on Herbal Medicine Use and Receiving Herbal Medicine Education in Jordan,” *Tropical Journal of Pharmaceutical Research* 16, no. 3 (2017): 689–96, Scopus, https://doi.org/10.4314/tjpr.v16i3.26. | Jordan |
| G. Gülpinar, “COMMUNITY PHARMACISTS’ PERSPECTIVES ON THE USE OF SOCIAL MEDIA OPINION LEADERS (INFLUENCER) IN THE MARKETING AND SALES OF FOOD SUPPLEMENTS AND HERBAL CONTENT PRODUCTS,” *Ankara Universitesi Eczacilik Fakultesi Dergisi* 47, no. 2 (2023): 1–10, https://doi.org/10.33483/jfpau.1247634. | Turkey |
| Y Sarinca et al., “Assessment of Consumer Perceptions in Ankara (Turkey) Toward Herbal Medicinal Products: A Survey Analysis in the Etimesgut District,” *CLINICAL AND EXPERIMENTAL HEALTH SCIENCES* 9, no. 1 (2019): 7–13, WOS:000462818200002, https://doi.org/10.5152/clinexphealthsci.2017.774. | Turkey |
| I. Gökkaya et al., “A Descriptive Survey Study in Endocrinology Clinic: Evaluation of the Use of Natural Products,” *CURRENT MEDICAL RESEARCH AND OPINION* 39, no. 11 (2023): 1439–49, https://doi.org/10.1080/03007995.2023.2270898. | Turkey |
| N.Y. Çeltek and U. Ünlü, “Traditional and Complementary Medicine Methods Which Have Become Widespread from the Perspective of Family Physicians and Self-Employed Pharmacists; Approaches, Thoughts,” *Eastern Journal of Medicine* 26, no. 1 (2021): 75–79, Scopus, https://doi.org/10.5505/ejm.2021.24482. | Turkey |
| N. Tarhan et al., “A Structural Equation Model for Pharmacy Students’ Acquiring Knowledge-Based Behavior towards Complementary and Alternative Medicine,” *European Journal of Integrative Medicine* 16 (2017): 54–60, Scopus, https://doi.org/10.1016/j.eujim.2017.10.007. | Turkey |
| G. Renda et al., “Pharmacy Students’ Knowledge and Attitudes about Homeopathy: A Descriptive Survey Conducted in Turkey,” *Homeopathy* 113, no. 1 (2022): 41–48, https://doi.org/10.1055/s-0043-1761267. | Turkey |
| HASAN HÜSEYİN EKER et al., *The Consumption Preferences on the Use of Herbal Products in Turkey and Opinions on Pharmacists*, 2017. | Turkey |
| S. Tezcan and M. Butur, “EVALUATION OF THE ATTITUDES AND PRACTICES OF PATIENTS REGARDING THE USE OF HERBAL PRODUCTS,” *Ankara Universitesi Eczacilik Fakultesi Dergisi* 46, no. 3 (2022): 817–26, https://doi.org/10.33483/jfpau.1122581. | Turkey |
| L. Yumrukaya et al., “Exploring the Dietary Supplement Use During COVID-19: Insights from Community Pharmacists, Pharmacy Staff, Academics, and Industry Experts,” *JOURNAL OF INTEGRATIVE AND COMPLEMENTARY MEDICINE*, ahead of print, July 24, 2024, https://doi.org/10.1089/jicm.2023.0632. | Turkey |
| Fatih Satil and Emine Aktaş, “Evaluation of using medicinal and aromatic plants by healthcare professionals during the coronavirus pandemic (COVID-19) period,” *KSÜ Tarim ve Doga Dergisi* 26, no. 2 (2023): 263–70, https://doi.org/10.18016/ksutarimdoga.vi.1105084. | Turkey |
| M. Sancar et al., “Assessment of the Attitude of Community Pharmacists and Pharmacy Technicians towards Diarrhea: A Simulated Patient Study in Turkey,” *Tropical Journal of Pharmaceutical Research* 14, no. 8 (n.d.): 1509–15. | Turkey |
| Vildan Özcan et al., “Measuring level of information and demand of users about the content of drug or food supplements,” *Türk Hijyen ve Deneysel Biyoloji Dergisi* 79, no. 2 (2022): 199–208, https://doi.org/10.5505/TurkHijyen.2022.12269. | Turkey |
| N. Tarhan and M. Arslan, “Determination of Factors Influencing Pharmacists While Recommending Immune-Enhancing Products via Analytic Hierarchy Process,” *TURKISH JOURNAL OF PHARMACEUTICAL SCIENCES* 19, no. 6 (2022): 701–5, https://doi.org/10.4274/tjps.galenos.2022.02686. | Turkey |
| Merve Memişoğlu and Gizem Otlatici, “The Safety of Herbal Medicines (Phytovigilance) from Community Pharmacists’ Perspective: A Cross-Sectional Study.,” in *Turkish Journal of Pharmaceutical Sciences*, vol. 19, no. 3, Istanbul, Galenos Publishing House, 2022. | Turkey |
| H. Basar Günes et al., “Assessment of Knowledge and Attitudes of Physicians and Pharmacists on Probiotics: A Cross-Sectional Survey,” *TURKISH JOURNAL OF PHARMACEUTICAL SCIENCES* 21, no. 1 (2024): 36–41, https://doi.org/10.4274/tjps.galenos.2023.36974. | Turkey |
| Naser Y Shraim et al., “Community Pharmacists’ Knowledge, Practices and Beliefs about Complementary and Alternative Medicine in Palestine: A Cross-Sectional Study,” *BMC Complementary and Alternative Medicine* 17 (2017): 1–12. | Palestine |
| R. Al-Ramahi et al., “Evaluation of Potential Drug-Herb Interactions among a Group of Palestinian Patients with Chronic Diseases,” *BMC Complementary and Alternative Medicine* 15, no. 221 (n.d.): (11 July 2015). | Palestine |
| ]Amer A. Koni et al., “Knowledge and Practice of Community Pharmacists Regarding the Safety of Drugs during Pregnancy: A Cross-Sectional Study from a Developing Country,” *BMC Pregnancy and Childbirth* 24, no. 1 (2024): 189, https://dx.doi.org/10.1186/s12884-024-06393-3. | Palestine |
| H. Naseef et al., “EXPLORING THE ROLE OF COMMUNITY PHARMACISTS IN DRUG INTERACTIONS: A CROSS-SECTIONAL STUDY IN PALESTINE,” *Bulletin of Pharmaceutical Sciences. Assiut* 46, no. 2 (2023): 1019–31, https://doi.org/10.21608/BFSA.2023.327571. | Palestine |
| Ramzi Shawahna, “Development of Key Performance Indicators for Capturing Impact of Pharmaceutical Care in Palestinian Integrative Healthcare Facilities: A Delphi Consensus Study,” *Evidence‐Based Complementary and Alternative Medicine* 2020, no. 1 (2020): 7527543. | Palestine |
| Ramzi Shawahna, “Quality Indicators of Pharmaceutical Care in Palestinian Integrative Healthcare Facilities: Findings of a Qualitative Study among Stakeholders,” *Evidence‐Based Complementary and Alternative Medicine* 2020, no. 1 (2020): 4520769. | Palestine |
| Waleed Sweileh et al., “Dispensing Practices, Attitudes and Knowledge of Pharmacists towards Herbal Products in Palestine,” *Ibnosina Journal of Medicine and Biomedical Sciences* 5, no. 03 (2013): 123–30. | Palestine |
| Maher Khdour et al., “Pharmacists’ Knowledge, Attitudes, and Practices towards Herbal Remedies in the West Bank: A Cross-Sectional Study,” *The Lancet* 391 (February 2018): S17, https://doi.org/10.1016/S0140-6736(18)30342-8. | Palestine |
| R. Al-Ramahi, “Patterns and Attitudes of Self-Medication Practices and Possible Role of Community Pharmacists in Palestine,” *International Journal of Clinical Pharmacology and Therapeutics* 51, no. 7 (2013): 562–67, Scopus, https://doi.org/10.5414/CP201814. | Palestine |
| ABDEL A Qawasmeh et al., “Pharmacists’ Knowledge and Perception Regarding Dietary Supplements in Palestine: Descriptive Study,” *Int J Pharm Pharm Sci* 12 (2020): 59–62. | Palestine |
| A. Abu Mhadi et al., “Exploring Medicines Use Patterns and Practices among the Public in the Gaza Strip, Palestine: A Qualitative Study,” *JOURNAL OF RESEARCH IN PHARMACY* 27, no. 1 (2023): 469–80, https://doi.org/10.29228/jrp.329. | Palestine |
| Mohammad Altamimi et al., “Knowledge, Attitudes and Practices Related to Dietary Supplements among a Group of Palestinian Pharmacists.,” *Sultan Qaboos University Medical Journal* 21, no. 4 (2021): 613–20, awr. | Palestine |
| Naseem Mohammed Abdulla et al., “Prevalence of, and Factors Associated with Health Supplement Use in Dubai, United Arab Emirates: A Population-Based Cross-Sectional Study,” *BMC Complementary and Alternative Medicine* 19 (2019): 1–11. | UAE |
| F. Naja et al., “Beyond Pillbox: A National Cross-Sectional Study on the Attitudes, Practices, and Knowledge of Community Pharmacists Regarding Complementary and Alternative Medicine,” *BMC COMPLEMENTARY MEDICINE AND THERAPIES* 24, no. 1 (2024), https://doi.org/10.1186/s12906-024-04391-8. | UAE |
| Ammar Abdulrahman Jairoun et al., “Development and Validation of a Tool to Improve Community Pharmacists’ Surveillance Role in the Safe Dispensing of Herbal Supplements,” *Frontiers in Pharmacology* 13, no. July (2022), https://doi.org/10.3389/fphar.2022.916223. | UAE |
| I. A. Basheti et al., “The Effects of Medications and the Roles of Pharmacists on the Recovery of Patients with COVID-19 Infection: An Epidemiological Study from the United Arab Emirates,” *HEALTHCARE* 11, no. 4 (2023), https://doi.org/10.3390/healthcare11040467. | UAE |
| Basheti et al., “The Effects of Medications and the Roles of Pharmacists on the Recovery of Patients with COVID-19 Infection: An Epidemiological Study from the United Arab Emirates.” | UAE |
| Osama H. Mohamed Ibrahim et al., “Evaluation of the Use and Attitudes of Pregnant and Postpartum Women towards Medicine Utilisation during Pregnancy in the United Arab Emirates: A National Cross-Sectional Study,” *International Journal of Clinical Practice* 75, no. 9 (2021): e14344, https://doi.org/10.1111/ijcp.14344. | UAE |
| Ibrahim Khalid Rayes and Omar Saad Saleh Abrika, “Community Pharmacists Knowledge and Perspectives Regarding the Medicinal Use of Nigella Sativa Seeds (Ranunculaceae): A Qualitative Insight from Dubai, United Arab Emirates,” *Journal of Medicinal Plants Research* 13, no. 19 (2019): 518–22. | UAE |
| N. M. Abdulla et al., “The Link between Knowledge and Practices in Relation to Herbal Supplement Use: For a Rapid Transfer of Knowledge in the Field of Phytovigilance and Pharmacovigilance Health Care Systems,” *JOURNAL OF PHARMACEUTICAL HEALTH SERVICES RESEARCH* 14, no. 2 (2023): 151–58, https://doi.org/10.1093/jphsr/rmad021. | UAE |
| A.A.A. Shamsher et al., “Herbal and Prescription Weight Loss Products: Awareness among Pharmacists about Efficacy and Safety,” *Journal of Pharmacy Practice and Research* 46, no. 4 (2016): 331–37, Scopus, https://doi.org/10.1002/jppr.1151. | UAE |
| I.K. Rayes et al., “Perception of Community Pharmacists toward Their Current Professional Role in the Healthcare System of Dubai, United Arab Emirates,” *Saudi Pharmaceutical Journal* 23, no. 3 (2015): 235–40, Scopus, https://doi.org/10.1016/j.jsps.2014.11.016. | UAE |
| A. S. Jarab et al., “Patterns of Drug-Related Problems and the Services Provided to Optimize Drug Therapy in the Community Pharmacy Setting,” *SAUDI PHARMACEUTICAL JOURNAL* 31, no. 9 (2023), https://doi.org/10.1016/j.jsps.2023.101746. | UAE |
| N. Nikvarz et al., “Medicinal Plants Used in Multiple Sclerosis Patients, Prevalence and Associated Factors: A Descriptive Cross-Sectional Study,” *BMC Complementary Medicine and Therapies* 24, no. 1 (2024), https://doi.org/10.1186/s12906-024-04587-y. | Iran |
| N. Keshvari et al., “Exploring Health Seeking Behaviors for Common Cold Management,” *EXPLORATORY RESEARCH IN CLINICAL AND SOCIAL PHARMACY* 11 (September 2023), https://doi.org/10.1016/j.rcsop.2023.100301. | Iran |
| G. Mehralian et al., “Client Priorities and Satisfaction with Community Pharmacies: The Situation in Tehran,” *International Journal of Clinical Pharmacy* 36, no. 4 (2014): 707–15, Scopus, https://doi.org/10.1007/s11096-014-9928-7. | Iran |
| F. Dabaghzadeh and R. Hajjari, “Practice of Community Pharmacists Related to Multivitamin Supplements: A Simulated Patient Study in Iran.,” in *International Journal of Clinical Pharmacy*, vol. 40, no. 1, Dordrecht, Springer, 2018; Salsabila Athirah Putri et al., “Sindrom Makan Malam, Asupan Serat, dan Pendapatan Rumah Tangga dengan Kejadian Gizi Lebih pada Siswa SMAN 6 Depok: Sindrom Makan Malam, Asupan Serat, dan Pendapatan Rumah Tangga dengan Kejadian Gizi Lebih pada Siswa SMAN 6 Depok,” *Amerta Nutrition* 7, no. 2SP (2023): 2SP, https://doi.org/10.20473/amnt.v7i2SP.2023.132-138. | Iran |
| G. Mehralian et al., “Knowledge, Attitude and Practice of Pharmacists Regarding Dietary Supplements: A Community Pharmacy-Based Survey in Tehran.,” *Iranian Journal of Pharmaceutical Research* 13, no. 4 (2014): 1457–65. | Iran |
| K. Mirnia et al., “Who Are the Main Recommenders of Herbal Drugs for Treating Neonatal Jaundice?,” *Journal of Comprehensive Pediatrics* 15, no. 1 (2024), https://doi.org/10.5812/jcp-140996. | Iran |
| Peivand Bastani et al., “Knowledge, Attitude and Practice of Iranian Pharmacists, Body Builders, and Their Coaches Regarding Sports Supplements,” *Journal of Research in Pharmacy Practice* 6, no. 3 (2017): 166–72. | Iran |
| Peivand Bastani et al., “Community Pharmacy-Based Survey on Pharmacists’ Knowledge, Attitude, and Performance Regarding Dietary Supplements: Evidence from South of Iran,” *National Journal of Physiology, Pharmacy and Pharmacology* 7, no. 4 (2017): 396–396, https://doi.org/10.5455/njppp.2017.7.1233518122016. | Iran |
| K. Danesh et al., “Knowledge, Attitude, and Practice (KAP) of Pharmacists about Natural Products Used for Urolithiasis in Shiraz, Iran,” *RESEARCH JOURNAL OF PHARMACOGNOSY* 10, no. 1 (2023): 5–14, https://doi.org/10.22127/rjp.2022.342725.1898. | Iran |
| F. Dabaghian et al., “Use of Medicinal Plants and Its Association with Health Literacy in the General Population of Iran During the COVID-19 Pandemy: A Web-Based Cross-Sectional Survey,” *RESEARCH JOURNAL OF PHARMACOGNOSY* 10, no. 1 (2023): 31–40, https://doi.org/10.22127/RJP.2022.366963.1997. | Iran |
| S.I. Mohammed and A.F.A. Al-Razaq, “Knowledge and Attitude of Iraqi Pharmacists in Baghdad Community toward Adverse Drug Reactions Monitoring,” *Asian Journal of Pharmaceutical and Clinical Research* 10, no. 4 (2017): 219–22, Scopus, https://doi.org/10.22159/ajpcr.2017.v10i4.16595. | Iraq |
| Samer I Mohammed et al., “Knowledge, Use and Recommendation of Iraqi Pharmacist toward Complementary and Alternative Medicine,” *Iraqi Journal of Pharmaceutical Sciences (P-ISSN 1683-3597 E-ISSN 2521-3512)* 29, no. 1 (2020): 88–93. | Iraq |
| H. F. Kasim, “Prevalence of Potential Drug Interactions among Outpatients’ Prescriptions of Community Pharmacies in Nineveh Governorate, Iraq,” *JOURNAL OF PHARMACY & PHARMACOGNOSY RESEARCH* 11, no. 2 (2023): 281–90, https://doi.org/10.56499/jppres23.1577_11.2.281. | Iraq |
| A. S. Mahdi et al., “A Statistical Study about Knowledge Pharmacy Students with Drugs Indicated and Contraindicated in Pregnancy,” *JOURNAL OF POPULATION THERAPEUTICS AND CLINICAL PHARMACOLOGY* 30, no. 3 (2023): E284–90, https://doi.org/10.47750/jptcp.2023.30.03.030. | Iraq |
| A. Karim Walaa et al., “Enhancing Adherence to Recommended Micronutrient Supplements during Pregnancy through Pharmacist Intervention,” *Kerbala Journal of Pharmaceutical Sciences مجلة كربلاء للعلوم الصيدلانية* 1, no. 22 (2023): 29–37. | Iraq |
| R. F. Hasan et al., “Knowledge, Attitude and Practice of Community Pharmacists on Medication Risks on Pregnant Women in Al Najaf City, Iraq,” *LATIN AMERICAN JOURNAL OF PHARMACY* 42, no. 4 (2023): 842–48. | Iraq |
| R. I. Nassar et al., “An Overview of the Experience of Iraqi COVID-19 Patients, and the Role of the Pharmacists during Their Infection,” *PHARMACY PRACTICE-GRANADA* 22, no. 3 (2024), https://doi.org/10.18549/PharmPract.2024.3.2994. | Iraq |
| A. H. Al-Hadi and E. M. Mikhael, “Pharmacists’ Perception about Efficacy, Safety, and Quality of Dietary Supplements That Used for Rheumatic Disorders in the Iraqi Pharmaceutical Market,” *PLOS ONE* 19, no. 7 (2024), https://doi.org/10.1371/journal.pone.0306380. | Iraq |
| S. K. Mohammed et al., *Medicine consumption edification in the framework of Iraqi consumer protection: An analysis study*, 1, no. 1 (2009): 46–69. | Iraq |
| Noura Issa Khram et al., “ASSESSMENT OF THE LEBANESE COMMUNITY PHARMACIST KNOWLEDGE, PRACTICE AND BARRIERS REGARDING THE PREVENTION OF OSTEOPOROSIS,” *BAU Journal - Health & Well-Being* 4, no. 2 (2022): 1–11, https://doi.org/10.54729/TJQN9288. | Lebanon |
| Mohamad Ali Hijazi et al., “The Role of Pharmacists in Complementary and Alternative Medicine in Lebanon: Users’ Perspectives,” *BMC Complementary Medicine and Therapies* 21 (2021): 1–10. | Lebanon |
| Samar Karout et al., “Assessing the Pharmaceutical Care Provision to Suspected COVID-19 Patients in Community Pharmacies: A Simulated Patient Study,” *BMC HEALTH SERVICES RESEARCH* 22, no. 467 (2022): (09 April 2022), https://doi.org/10.1186/s12913-022-07870-1. | Lebanon |
| M. A. Hijazi et al., “Beliefs, Practices and Knowledge of Community Pharmacists Regarding Complementary and Alternative Medicine: National Cross-Sectional Study in Lebanon.,” in *BMJ Open*, vol. 9, no. 3, London, BMJ Publishing Group, 2019. | Lebanon |
| N. Makkaoui et al., “Knowledge, Attitudes, and Practices Regarding Drug Interactions among Community Pharmacists,” *Journal of Public Health (Germany)* 29, no. 6 (2021): 1357–63, Scopus, https://doi.org/10.1007/s10389-020-01252-9. | Lebanon |
| M.A. Hijazi et al., “Complementary and Alternative Medicine Use during the COVID-19 Pandemic: Community Pharmacists’ Knowledge, Attitudes, and Practices,” *Research in Social and Administrative Pharmacy* 19, no. 3 (2023): 502–9, Scopus, https://doi.org/10.1016/j.sapharm.2022.10.009. | Lebanon |
| M. Assi et al., “Herbal Medicine Use among Adult Lebanese Patients with Chronic Diseases: A Descriptive Cross-Sectional Study,” *Open Public Health Journal* 17 (2024), https://doi.org/10.2174/0118749445293046240329072634. | Lebanon |
| Aya Elsayed Abdelwahed et al., “Prevalence and Influencing Factors of Self-Medication during the COVID-19 Pandemic in the Arab Region: A Multinational Cross-Sectional Study,” *BMC Public Health* 23, no. 180 (2023), https://doi.org/10.1186/s12889-023-15025-y. | Multiple Arab Countries |
| Joanna E. Harnett et al., “Defining and Supporting a Professional Role for Pharmacists Associated with Traditional and Complementary Medicines: A Cross-Country Survey of Pharmacists,” *Frontiers in Pharmacology* 14, no. August (2023), https://doi.org/10.3389/fphar.2023.1215475. | Multiple Arab Countries |
| N.A. Alshehab et al., “Evaluating Pharmacists’ Knowledge, Attitude, and Practices toward Amiodarone Cross-Reactivity with Iodine: A Cross-Sectional Pilot Study,” *PeerJ* 10 (2022), Scopus, https://doi.org/10.7717/peerj.13665. | Multiple Arab Countries |
| Iman A. Basheti et al., “Patient Perspectives of the Role of the Community Pharmacist in the Middle East: Jordan, United Arab Emirates and Iraq,” *Pharmacology &amp; Pharmacy* 5, no. 6 (2014): 6, https://doi.org/10.4236/pp.2014.56069. | Multiple Arab Countries |
| K. Iskandar et al., “Highlighting the Pivotal Role of the Pharmacist in Influencing Health Behaviours during Emergency Crisis: A Lesson from the COVID-19 Pandemic,” *Pharmacy Education* 23, no. 1 (2023): 676–92, https://doi.org/10.46542/pe.2023.231.676692. | Multiple Arab Countries |
| Sahar A Fahmy et al., “Pharmacists’ Attitude, Perceptions and Knowledge towards the Use of Herbal Products in Abu Dhabi, United Arab Emirates,” *Pharmacy Practice* 8, no. 2 (2010): 109. | Multiple Arab Countries |
| A. S. Ghaith, “Population Perceptions and Views of Community Pharmacists during the Covid-19 Pandemic: A Comparative, Cross-Sectional Study in Jordan and Kuwait,” *Regional Statistics* 14, no. 4 (2024): 793–812, https://doi.org/10.15196/RS140408. | Multiple Arab Countries |
| A. Awad and D. Al-Shaye, “Public Awareness, Patterns of Use and Attitudes toward Natural Health Products in Kuwait: A Cross-Sectional Survey.,” *BMC Complementary and Alternative Medicine* 14, no. 105 (2014): (19 March 2014). | Kuwait |
| Abdelmoneim Awad and Mohammad Waheedi, “Community Pharmacists Role in Obesity Treatment in Kuwait: A Cross-Sectional Study,” *BMC Public Health* 12 (2012): 1–9. | Kuwait |
| Abdullah Albassam and Abdelmoneim Awad, “Community Pharmacists’ Services for Women during Pregnancy and Breast Feeding in Kuwait: A Cross-Sectional Study.,” *BMJ Open* (England) 8, no. 1 (2018): e018980, Ovid MEDLINE(R) <2018>, https://doi.org/10.1136/bmjopen-2017-018980. | Kuwait |
| A. I. Awad et al., “Knowledge, Perceptions and Attitudes toward Complementary and Alternative Therapies among Kuwaiti Medical and Pharmacy Students,” *Medical Principles and Practice: International Journal of the Kuwait University, Health Science Centre* 21, no. 4 (2012): 350–54, https://doi.org/10.1159/000336216. | Kuwait |
| Nada A Abahussain et al., “Pharmacists’ Attitudes and Awareness towards the Use and Safety of Herbs in Kuwait,” *Pharmacy Practice (Internet)* 5, no. 3 (2007): 125–29. | Kuwait |
| F.N. Hussain et al., “Association of Pharmacy Students’ Cultural Beliefs with Perceived Knowledge, Beliefs, Confidence, and Experience with Complementary Medicine,” *Currents in Pharmacy Teaching and Learning* 13, no. 2 (2021): 159–63, Scopus, https://doi.org/10.1016/j.cptl.2020.09.009. | Qatar |
| Nadir Kheir et al., “Pharmacists’ Knowledge and Attitudes about Natural Health Products: A Mixed-Methods Study,” *Drug, Healthcare and Patient Safety*, Taylor & Francis, 2014, 7–14. | Qatar |
| David Mottram et al., “Perspective of Pharmacists in Qatar Regarding Doping and Anti-Doping in Sports.,” *The Journal of Sports Medicine and Physical Fitness* (Italy) 56, no. 6 (2016): 817–24, Ovid MEDLINE(R) <2016>. | Qatar |
| Sawsan AlMukdad et al., “Exploring the Role of Community Pharmacists in Obesity and Weight Management in Qatar: A Mixed-Methods Study.,” *Risk Management and Healthcare Policy* (England) 14, no. 101566264 (2021): 2771–87, Ovid MEDLINE(R) PubMed-not-MEDINE <2021>, https://doi.org/10.2147/RMHP.S309142. | Qatar |
| M. Dumenci et al., “The Attitude, Knowledge, and Evaluation of Herbal Medicinal Products for Respiratory Diseases in Northern Cyprus among Pharmacists, Patients, and Pulmonologists,” *INDIAN JOURNAL OF PHARMACEUTICAL EDUCATION AND RESEARCH* 57, no. 4 (2023): 1242–50, https://doi.org/10.5530/ijper.57.4.148. | Cyprus |
| E Tülek et al., “Herbal Medicines Use in Pregnancy in Northern Cyprus,” *JOURNAL OF PHARMACEUTICAL RESEARCH INTERNATIONAL* 33, no. 43A (2021): 487–504, WOS:000695212400026, https://doi.org/10.9734/JPRI/2021/v33i43A32515. | Cyprus |
| H. K. Sakalli et al., “Attitudes and Approaches towards COVID-19 Management among the Public and Pharmacists in Northern Cyprus,” *Tropical Journal of Pharmaceutical Research* 21, no. 6 (2022): 1309–16, https://doi.org/10.4314/tjpr.v21i6.25. | Cyprus |
| Hoda Fathy et al., “Attitudes, Knowledge and Practice of Herbal Remedy Use among the Population Visiting Pharmacies and Health Care Providers in Alexandria, Egypt: A Cross-Sectional Study,” *European Journal of Medicinal Plants* 30, no. 2 (2019): 1–10. | Egypt |
| M. Medhat et al., “Knowledge, Attitude and Practice of Community Pharmacists towards Nutrition Counseling.,” in *International Journal of Clinical Pharmacy*, vol. 42, no. 6, Dordrecht, Springer, 2020. | Egypt |
| Sally Ezzat, “A Study of the Use of Drugs in the Treatment of Obesity among Adult Females.,” *International Journal of Health Care Quality Assurance* (England) 25, no. 8 (2012): 730–41, Ovid MEDLINE(R) <2012>, https://doi.org/10.1108/09526861211270668. | Egypt |
| Sujith Haridass et al., “Knowledge, Attitude, and Use of Complementary and Alternative Medicine (CAM) among Pharmacy and Nursing Students in Oman,” *International Journal of Advanced Community Medicine* 1, no. 1 (2018): 40–43, https://doi.org/10.33545/comed.2018.v1.i1a.9. | Oman |
| Anas Younis Duraz and Shah Alam Khan, “Knowledge, Attitudes and Awareness of Community Pharmacists Towards the Use of Herbal Medicines in Muscat Region.,” *Oman Medical Journal* 26, no. 6 (2011): 451–53, awr. | Oman |
| L. Al-Qysi et al., “Investigating the Characteristics of Probiotics Marketed in the Middle East and Pharmacists’ Perception of Use in Muscat, Oman,” *PharmaNutrition* 13 (2020), Scopus, https://doi.org/10.1016/j.phanu.2020.100202. | Oman |
| Ali Hiba Hashim Mohamed et al., “Knowledge, Attitudes, and Practice of Self-Medication among Medical Students at Sudan International University, Sudan,” *Al-Kindy College Medical Journal مجلة كلية الطب الكندي* 19, no. 1 (2023): 57–61. | Sudan |
| Amah Alsaid et al., “Knowledge, Attitude and Practice of Community Pharmacists towards Food-Drug Interactions; A Prospective Cross-Sectional Analysis from Sudan,” *LATIN AMERICAN JOURNAL OF PHARMACY* 41, no. 3 (2022): 506–14. | Sudan |
| E. A. Saleh et al., “Changes in Drug Demand When a Pandemic Coincides with Other Outbreaks in a War Zone Country: A Cross-Sectional Pilot Study,” *JOURNAL OF PHARMACEUTICAL POLICY AND PRACTICE* 15, no. 1 (2022), https://doi.org/10.1186/s40545-022-00487-z. | Yemen |
| AA Thabit et al., “Perception of Community Pharmacists and Pharmacy Technicians in Sanaa-Yemen about Risks of Use of Herbal Products during Pregnancy and Breastfeeding,” *World J. Pharm. Res* 9, no. 4 (2020): 1707–16. | Yemen |
| R. I. Nassar et al., “COVID-19 Infected Patients’ Experiences in Syria, and the Role of the Pharmacists during Their Infection,” *PHARMACY PRACTICE-GRANADA* 20, no. 1 (2022), https://doi.org/10.18549/PharmPract.2022.1.2617. | Syria |
| S. A. Rabbani et al., “A Systematic Scoping Review of Implementation of Telepharmacy during COVID-19,” *Journal of Applied Pharmaceutical Science* 13, no. 4 (2023): 30–44, https://doi.org/10.7324/JAPS.2023.113646. | A review, so NA |
| C. O. L. Ung et al., “Pharmacy Education in Traditional and Complementary Medicines - A Systematic Review,” *RESEARCH IN SOCIAL & ADMINISTRATIVE PHARMACY* 19, no. 10 (2023): 1331–53, https://doi.org/10.1016/j.sapharm.2023.07.007. | A review, so NA |
