# Supplementary material for: Role of community pharmacists in the safe and effective use of complementary and alternative medicine in the Middle East: A scoping review
Source: PLoS One. 2025 Sep 26;20(9):e0332932. doi: 10.1371/journal.pone.0332932 (PMC12469160; doi:10.1371/journal.pone.0332932)
Supplement: Attachment — Submitted filename: Response to Reviewers.docx [file pone.0332932.s005.docx]

7/20/2025

Rebuttal letter

Dear Editor and members of the review board,

We thank you and the reviewers for the thoughtful and constructive feedback provided on our manuscript. We have carefully revised the manuscript in response to the comments and have addressed each point in detail below. The suggestions greatly contributed to improving the clarity, rigor, and relevance of our work. Below, we provide a point-by-point response outlining the changes made in the revised version.

Editor’s comments:

1. *Limited Diversity in Study Design Across Included Studies: While the scoping review is comprehensive, nearly all of the included studies employed cross-sectional designs (98.06%). This methodological homogeneity limits the depth of insight into causality, change over time, or the impact of interventions related to CAM practices. The authors should: a) More explicitly acknowledge this limitation in the discussion and recommend that future studies explore longitudinal, experimental, or mixed-methods designs to advance the field. b) Consider stratifying findings by study design type (e.g., observational vs. interventional) to assess whether recommendations differ depending on methodological rigor*.

**Authors’ response**: We thank the editor for his comment and suggestions. Accordingly, we have revised the limitation section to include the following text: Another limitation to this review, especially to the thematic analysis section, is the fact that most of the studies identified were of cross-sectional nature. This limits the ability to draw conclusions about causality, temporal trends, or the effectiveness of interventions related to CAM practices. Future research should prioritize longitudinal, experimental, and mixed-methods designs to provide deeper insights into the dynamics, outcomes, and contextual influences of CAM use. (Lines 462-466)

The suggestion of the editor to stratify by research design (cross sectional versus interventional) is surely a key approach to limit the impact of the cross-sectional design on the findings of this review. In this review, the fact that only one study had an intervention design hindered our capability to carry out a stratified analysis.

2. *Lack of Visual Representation of Thematic Synthesis: Although the manuscript notes a thematic synthesis using NVivo, there is no visible presentation of how themes and subthemes were derived (e.g., thematic map, concept framework, or coding tree). Please include a visual figure (e.g., thematic map or framework) showing the analytical process from raw codes to themes. This would enhance transparency and help readers understand how conclusions were drawn from the data.*

**Authors’ response**: We thank the reviewer for this insightful suggestion, which indeed helps improve the transparency and clarity of our thematic analysis. In response, we have revised figure 3 to include a **thematic map** that visually represents the analytical process the overarching themes to the individual sub themes. This revision illustrates how data were interpreted and organized during the synthesis process and complements the narrative description in the Methods and Results sections.

3. *Absence of Quality Assessment or Risk of Bias Appraisal: Although scoping reviews typically do not require formal risk of bias assessments, the authors could improve transparency by a) briefly commenting on the overall methodological quality of included studies (e.g., through a descriptive overview of sample sizes, response rates, or validation processes), b) Consider discussing whether any quality trends (e.g., lack of validated tools or unreported response rates) might affect the robustness of findings and recommendations, c) At minimum, justify why quality appraisal was not conducted and explain how this affects interpretation*.

**Authors’ response**: In line with the editor’s comment, the discussion and limitations sections of the manuscript were revised, and the following paragraphs were added:

- Discussion: Although the primary aim of scoping reviews is to map the extent and nature of existing evidence rather than to assess study quality, certain methodological concerns warrant attention. Beyond the predominance of cross-sectional designs and the lack of longitudinal and interventional studies, our review identified additional limitations in research rigor. Notably, only 60% of the studies reported using validated tools or pilot-testing their questionnaires, which raises concerns about the reliability of the data collected. Furthermore, only 75% of the studies indicated obtaining Institutional Review Board (IRB) approval, and 55% did not include any funding statement, limiting transparency and potentially introducing bias. These findings highlight the need for more robust methodological standards in future research on CAM practices in pharmacy practice. (Lines 409-417)
- Limitations: Additionally, the findings of this scoping review are inherently limited by the quality of the included studies. Methodological shortcomings—such as the limited use of validated tools, inconsistent reporting of ethical approval, and lack of funding disclosure—may affect the reliability and generalizability of the reported outcomes. (Lines 466-469)

*4. Inconsistent Handling of Terminology (e.g., "Middle East"): The use of "Middle East" (ME) is not explicitly defined in terms of which countries were included. While a country list appears in the results section, it should be clearly stated earlier (e.g., in the methods or inclusion criteria). Please define the scope of "Middle East" explicitly, possibly with reference to WHO or geopolitical classifications.*

**Authors’ response**: As recommended by the reviewer, we have revised Methods section to include the following text: For the purpose of this review, the Middle East was defined based on a geopolitical framework commonly adopted by the United States and NATO, encompassing the following countries: Bahrain, Cyprus, Egypt, Iran, Iraq, Israel, Jordan, Kuwait, Lebanon, Oman, Palestine (Gaza and West Bank), Qatar, Saudi Arabia, Syria, Turkey, and the United Arab Emirates. This definition was selected to reflect both regional proximity and strategic relevance in international policy and health discourse. (Lines 130-135)

*5. Also, consider adding a supplementary table listing the included studies by country to enhance clarity and transparency.*

**Authors’ response**: As recommended by the editor, a table outlining the country and the corresponding studies is added as supplementary material to the revised submission. (Appendix C)

*6. Insufficient Detail on Limitations of Narrative Synthesis: The limitations section acknowledges the use of narrative synthesis but does not elaborate on the specific interpretive challenges this presents. The authors should clearly explain the implications of using narrative synthesis for a dataset with heterogeneous study aims, tools, and outcomes. Acknowledge that subjective interpretation could bias the conclusions, even with dual-coding and software support.*

**Authors’ response**: As recommended by the reviewer, the limitation section was revised to emphasize the limitations of the narrative synthesis, as follows: A limitation of this review is that the results were presented narratively due to the considerable heterogeneity of the analyzed literature in terms of aim, scope, population, and research methods. While narrative synthesis allowed for a broad and flexible interpretation of findings, it also carries inherent interpretive challenges. In particular, synthesizing data from studies with diverse objectives, tools, and outcome measures may limit comparability and introduce subjective interpretation. Although potential bias was mitigated through the involvement of two independent reviewers and the use of two distinct analytic approaches (manual review and NVivo 20 software), the interpretive nature of narrative synthesis remains a source of possible bias that readers should consider. (Lines 454-462)

*7. Underdeveloped Discussion of Impact Factor Trends: The manuscript presents an interesting finding on the increasing volume of publications but stagnating journal impact factors. However, this point is underexplored. The discussion would benefit from a more critical interpretation: What does this trend imply about scientific visibility, research quality, or regional research funding? Are papers being published in lower-impact journals due to limited collaboration, lack of interventional studies, or methodological weaknesses?*

**Authors’ response**: In response to the reviewer’s suggestion, an additional paragraph has been incorporated into the revised manuscript to provide a more in-depth discussion of the observed trend in journal impact factor, as follows: This stagnation in journal impact factor, despite rising publication volume, may also reflect broader challenges in the region's research ecosystem. These include limited cross-border or international collaborations, a predominance of observational or descriptive designs with fewer interventional or longitudinal studies, and methodological shortcomings that may reduce the likelihood of acceptance in higher-impact journals. Furthermore, structural issues such as constrained research funding, limited access to research infrastructure, and underinvestment in training and capacity-building may also contribute to this trend. Collectively, these factors may hinder the visibility, scientific influence, and citation potential of Middle Eastern research, underscoring the importance of policy-level investment in research quality, mentorship, and global engagement. (Lines 360-381)

*8. No Explicit Link Between Themes and Policy Recommendations: The paper outlines strong thematic categories (opportunities, challenges, recommendations) but stops short of aligning these directly with practical or policy-level interventions. Consider including a concise table that maps each theme (e.g., knowledge gaps, poor regulation) to specific, evidence-based recommendations for policy, education, or clinical practice.*

**Authors’ response**: As recommended by the reviewer, figure 3 was revised to align the challenges to the recommendations in terms of education, practice and policy and regulations.

Reviewer #1: General Comments

1. *The discussion acknowledges repeated themes across studies. While this reinforces consistency, the manuscript would benefit from more emphasis on gaps that remain under-explored (e.g., digital health integration, policy implementation success stories). Consider adding a visual or tabular summary highlighting under-researched countries or topics within the region.*

**Authors’ response**: We appreciate the reviewer’s insightful comment regarding the need to emphasize under-explored gaps. In the revised manuscript, we have expanded the discussion to highlight areas that remain insufficiently addressed in the literature, particularly the integration of digital health tools in CAM pharmacy practice and the lack of documented policy implementation success stories, as follows: In addition, the review highlighted a striking lack of exploration into emerging domains such as the integration of digital health technologies in CAM pharmacy practice and the evaluation of successful policy implementation cases—both of which are essential to advancing the field in line with global healthcare innovations. (Lines 374-377)

As recommended by the reviewer, Table 2 was added to summarize the gaps that were identified in the reviewed research papers. In this table, and in line with a subsequent comment of the reviewer, a column was added to present practical recommendations for future research.

2. *The process of thematic analysis is briefly described but lacks depth. Clarify how inter-rater reliability was ensured and how themes were validated*.

**Authors’ response**: We appreciate the reviewer’s suggestion. In the revised manuscript, we expanded the description of the thematic analysis process to clarify how coding consistency was established through a pilot phase, how discrepancies were resolved by consensus, and how final themes were validated through team discussions. These additions highlight the measures taken to ensure inter-rater reliability and analytical rigor. (Lines 190-198; 200-202)

*3. The PRISMA flowchart is referenced but missing ("Error! Reference source not found."). Ensure figures and tables are properly inserted and labeled.*

**Authors’ response**: We apologize for this technical error. The document was fixed to clearly show the figure of the PRISM flowchart.

*4. The manuscript relies heavily on textual description. Consider condensing parts of the results into summary tables (e.g., summary of challenges per country or per theme).*

**Authors’ response**: As recommended by the reviewer, the results section was revised and condensed.

*5. Some statistics are over-detailed (e.g., breakdowns of questionnaire validation); grouping similar categories could improve readability*.

**Authors’ response**: As recommended by the reviewer, table 1 was revised and many descriptive variables were regrouped (Language, study design, type of qualitative study, collaborations, pilot test of the questionnaire). The revised Table 1 is now a more concise and clearer version.

*6. The language is generally academic, but some paragraphs are overly verbose or redundant. Example: lines 377–386 and 439–451 can be condensed without losing meaning.*

**Authors’ response**: We thank the reviewer for raising this issue, the text of the manuscript, including the Results and Discussion sections, was revised and condensed.

*7. Minor grammatical errors (e.g., "it is wide use" should be “its wide use”).*

**Authors’ response**: The text was revised as per the reviewer’s recommendation

*8. While the recommendations are well-presented, more concrete suggestions for implementation would strengthen the practical value of the review.*

**Authors’ response**: As recommended by the reviewer, Table 2 in the discussion was revised to include a column presenting practical recommendations for future research, as follows:

| **Aspect of Research** | **Identified Gaps** | **Practical Recommendations** |
| --- | --- | --- |
| **Study design** | Predominance of cross-sectional surveys; limited intervention and cohort studies | Encourage the use of longitudinal, interventional, and mixed methods designs to assess outcomes over time and the impact of specific programs |
|  | Limited qualitative research | Promote in-depth qualitative and mixed-methods research to capture contextual insights and lived experiences of pharmacists and CAM users |
| **Study tools** | Limited use of validated research instruments | Develop and adopt standardized, validated tools across studies |
| **Study themes** | Repetitive topics; lack of innovation; under-exploration of digital technologies and policy implementation outcomes | Prioritize gap analysis during research planning; promote studies that explore digital health integration, telepharmacy, AI tools, and evaluations of real-world policy implementation |
| **Collaborations** | Limited international and cross-sectoral collaborations | Strengthen inter sectoral partnerships to support multidisciplinary, cross-border projects |
| **Ethical considerations** | A sizeable proportion of studies did not report ethical approval | Require IRB approval for all studies involving human subjects as a standard practice |
| **Funding** | Constrained and inconsistently reported funding | Advocate for national and regional funding initiatives for CAM and pharmacy research; encourage greater transparency in reporting funding sources |

*9. It is advisable to distinguish between short-term and long-term recommendations for policymakers and educators.*

**Authors’ response**: We thank the reviewer for this suggestion. Accordingly, the conclusion was revised to outline short- and long-term actionable recommendations, as follows:

This review presents a comprehensive examination of the literature on the roles of CPs related to CAM in the ME region. The analysis reveals growing research activity, though characterized by repetitive approaches and limited collaboration. These findings provide a foundation for developing an evidence-informed research agenda that addresses overlooked areas, promotes innovative methodologies—including interventional, qualitative, mixed, and longitudinal designs—and fosters collaboration among academia, the pharmacy and CAM sectors, and NGOs. In the short term, policymakers and educators should integrate CAM content into pharmacy curricula, expand continuing education, and improve access to validated tools and reliable information. Long-term strategies should focus on establishing national practice guidelines, strengthening regulatory frameworks, formalizing CAM service models in pharmacies, and supporting cross-sectoral research infrastructure and global collaboration. These short- and long-term actions offer a roadmap to enhance educational, regulatory, and professional aspects of CAM pharmacy practice. The evidence presented can support researchers, policymakers, educators, and healthcare professionals in optimizing the contribution of CPs to the safe and effective use of CAM across the Middle East. (Lines 470-482)

*10. Line 50: Consider stating the countries covered rather than just “ME region”.*

**Authors’ response**: This comment was also raised by the editor and was answered as follows:

“As recommended, we have revised Methods section to include the following text: For the purpose of this review, the Middle East was defined based on a geopolitical framework commonly adopted by the United States and NATO, encompassing the following countries: Bahrain, Cyprus, Egypt, Iran, Iraq, Israel, Jordan, Kuwait, Lebanon, Oman, Palestine (Gaza and West Bank), Qatar, Saudi Arabia, Syria, Turkey, and the United Arab Emirates. This definition was selected to reflect both regional proximity and strategic relevance in international policy and health discourse. (Lines 130-135)

*11. Line 78: “desire to be actively engaged in their health decision-making” – clarify or simplify phrasing.*

**Authors’ response**: The text was revised to read: “desire to take an active role in their healthcare decisions”.

*12. Line 289: Correct “who reporting” to “who reported”.*

**Authors’ response**: The text was revised as per the reviewer’s recommendation.

*13. Line 308: Consider rewording for clarity: “restrains their daily practices across several ME countries”.*

**Authors’ response**: As per the reviewer’s recommendation, the sentence was revised to read: “….as a result, limits their ability to carry out daily practice effectively”

We hope that we have addressed the comments and suggestions of the reviewers, and we look forward to a favorable decision from the journal regarding our revised submission.

Best regards

On behalf of the authors

Mohamad Hijazi
